# Magnetic resonance spectroscopy as marker for neurodegeneration in X-linked adrenoleukodystrophy

**DOI:** 10.1016/j.nicl.2021.102793

**Published:** 2021-08-24

**Authors:** Stephanie I.W. van de Stadt, Anouk Schrantee, Irene C. Huffnagel, Wouter J.C. van Ballegoij, Matthan W.A. Caan, Petra J.W. Pouwels, Marc Engelen

**Affiliations:** a Department of Pediatric Neurology, Emma Children’s Hospital, Amsterdam University Medical Centers, Amsterdam, The Netherlands; b Department of Radiology and Nuclear Medicine, Amsterdam University Medical Centers, Amsterdam, The Netherlands; c Department of Neurology, OLVG Hospital, Amsterdam, The Netherlands; d Department of Biomedical Engineering & Physics, Amsterdam University Medical Centers, University of Amsterdam, Amsterdam, The Netherlands

**Keywords:** X-linked adrenoleukodystrophy, Magnetic Resonance Spectroscopy, Myelopathy, Neurodegeneration, MRI, Imaging biomarker

## Abstract

X-linked adrenoleukodsytrophy (ALD) is a genetic neuro-metabolic disorder, causing a slowly progressive myelopathy in adult male and female patients. New disease modifying therapies for myelopathy are under development. This calls for new (imaging) markers able to measure disease severity and progression in clinical trials. In this prospective cohort study, we measured cerebral metabolite levels with Magnetic Resonance Spectroscopy (MRS), and evaluated their potential as biomarkers for disease severity and neurodegeneration in ALD. We used a comprehensive protocol of 3T Magnetic Resonance Spectroscopic Imaging (MRSI) and 7T Single Voxel Spectroscopy (SVS) in a large cohort of adult ALD males without cerebral demyelination. One hundred seven baseline scans – 59 obtained in ALD patients (42 3T MRSI and 17 7T SVS) and 48 obtained in healthy male controls (32 3T MRSI and 16 7T SVS) – and 82 one and two-year follow-up scans (66 3T MRSI and 16 7T SVS) of ALD patients were included. Both protocols showed significantly lower concentration ratios of N-acetylaspartate/creatine (tNAA/tCr) and Glx (glutamine + glutamate)/tCr in the grey and white matter of patients, compared to controls. A novel finding is the higher level of inositol (Ins)/tCr and choline containing compounds (tCho)/tCr in ALD patients without cerebral demyelination. Furthermore, tNAA/tCr correlated strongly with clinical measures of severity of myelopathy. There was no detectable change in metabolite ratios after one-year or two-year follow-up. Our results imply that cerebral metabolite levels – and more specifically the tNAA/tCr ratio – measured with MRS, have potential value as (imaging) biomarkers in ALD.

## Highlights

• Spectroscopy provides valuable biomarker information for X-linked adrenoleukodystrophy.
• Reduced metabolite levels indicate axonal damage in the brain of patients with ALD.
• Two methods for spectroscopy, acquired at different field strengths, provide similar results.

## 1 Introduction

Adrenoleukodsytrophy (ALD) is an X-linked metabolic disorder, caused by mutations in the *ABCD1* gene (Moser et al., 2001, Kemp et al., 2016). Mutations cause dysfunction of ALD protein, a peroxisomal transmembrane protein, resulting in impaired β-oxidation of very long chain fatty acids (VLCFA) and elevated levels of VLCFAs in plasma and tissues (Singh et al., 1984, Mosser et al., 1994). In males, there is a broad spectrum of symptoms, from isolated adrenocortical insufficiency to a rapidly progressive cerebral demyelination (cerebral ALD) (Van Geel et al., 2001, Huffnagel et al., 2019). Eventually, all men – and most women – will develop a slowly progressive and invalidating myelopathy (Huffnagel et al., 2019, Huffnagel et al., 2019). Currently, treatment for myelopathy is supportive only, but new potential therapies are emerging. There is a great need for identification of surrogate outcome measures or (imaging) biomarkers able to monitor disease severity and progression in clinical trials, as current clinical outcome measures require large numbers of patients and long follow-up (Huffnagel et al., 2019).

An imaging technique that provides insight in the brain pathology of ALD is Magnetic Resonance Spectroscopy (MRS). MRS is used to measure cerebral metabolite levels, such as N-acetylaspartate (tNAA, including contributions of N-acetyl aspartyl glutamate), choline containing compounds (tCho), inositol (Ins), glutamate (Glu), glutamine (Gln), lactate (Lac) and creatine (tCr). In all ALD patients – including heterozygotes, cerebral ALD and asymptomatic patients – significantly reduced tNAA levels and correspondingly lower tNAA/tCr ratios have been found compared to controls (Pouwels et al., 1998, Fatemi et al., 2003, Rajanayagam et al., 1996, Dubey et al., 2005, Eichler et al., 2002). A reduction in tNAA levels is an indicator of axonal damage, which is the pathological hallmark of the myelopathy in ALD (Powers et al., 2000, Powers et al., 2001), but occurs in cerebral ALD as well. Additionally, in cerebral ALD, concentrations of tCho, Lac and Ins were elevated, which corresponds to the process of active demyelination (Eichler et al., 2002, Kruse et al., 1994, Ratai et al., 2008).

MRS can be acquired at varying spatial coverage and field strength. Single-voxel spectroscopy (SVS) may be superior for diseases in which the affected brain region is well defined, as the measured area is smaller and more accessible to analyze. Magnetic Resonance Spectroscopic Imaging (MRSI), in which data from multiple voxels within a whole area or “slab” are obtained, is more suitable for studying diffuse disease processes (Öz et al., 2014). With ultra-high magnetic field strengths (7T) sensitivity of metabolite concentration measurements increases due to increased signal-to-noise ratio (SNR) and spectral resolution, which allows for better separation of individual metabolite peaks. However, this increased sensitivity might be attenuated by enhanced susceptibility artefacts. These characteristics make both 3T and 7T MRS of potential interest to include.

The majority of previous ALD studies on MRS focused on patients with cerebral ALD and consisted of small numbers of patients. Furthermore, longitudinal data is often lacking. In this prospective study we evaluated the potential of cerebral metabolite levels, measured with MRS, as imaging biomarkers for disease severity in ALD. We used a comprehensive protocol of 3T MRSI and 7T SVS in a large cohort of adult ALD males without cerebral involvement, and monitored disease severity over a 2-year follow-up period.

## 2 Methods

### 2.1 Patient selection

This study was part of a prospective cohort study (“the Dutch ALD cohort”) performed at Amsterdam University Medical Centers (location AMC, Amsterdam, The Netherlands), the national referral center for ALD, between June 2015 and February 2018. Clinical data of this cohort has been published previously (Huffnagel et al., 20192019). For this particular study we included all male patients over 16 years of age with confirmed diagnosis of ALD (VLCFA and *ABCD1* analysis). Exclusion criteria were cerebral ALD (defined as gadolinium enhancing and non-enhancing cerebral white matter lesions on MRI), other neurological diseases interfering with the assessment of myelopathy, or contra-indications for MRI examination. Additionally, for 7T MR imaging patients were eligible to participate if they were over 18 years of age and if there were no additional contra-indications for 7T MRI examination (such as devices compatible with 3T but not 7T imaging). The control group consisted of age-matched healthy male individuals without clinical evidence of neurological disease. For 7T MR imaging a subset of the healthy controls were included. Written informed consent was obtained from all participants. The study protocol was approved by the local Institutional Review Board (METC 2014_347).

### 2.2 Clinical assessment

History and neurological examination were assessed at baseline and at one-year and two-year follow-up, as described previously (Huffnagel et al., 20192019). Based on history and neurological examination, patients were classified as symptomatic or asymptomatic. Symptomatic patients were defined as having signs and symptoms of myelopathy (Huffnagel et al., 2019, Engelen et al., 20142014). Three outcome measures were used to assess severity of myelopathy: Expanded Disability Status Scale (EDSS), Severity Scoring system for Progressive Myelopathy (SSPROM) and Timed Up-and-Go (TUG). The EDSS measures neurological disability and ranges from 0 (no disability) to 10 (death) (Kurtzke and -, , 1444.). The SSPROM scores symptoms of myelopathy and ranges from 0 to 100, with lower scores indicating a higher degree of impairment (Castilhos et al., 2012). TUG measures the time to get up from a chair, walk 3 m, walk back and sit down again (Podsiadlo and Richardson, 1991). Furthermore, semi-quantitative measurements of vibration sense were performed with a Rydel-Seiffer tuning fork at the hallux and internal malleolus. The mean score of vibration sense at the left and right hallux and internal malleolus (vibration score foot) was used for analysis.

### 2.3 MRI protocols

**Fig. 1:**
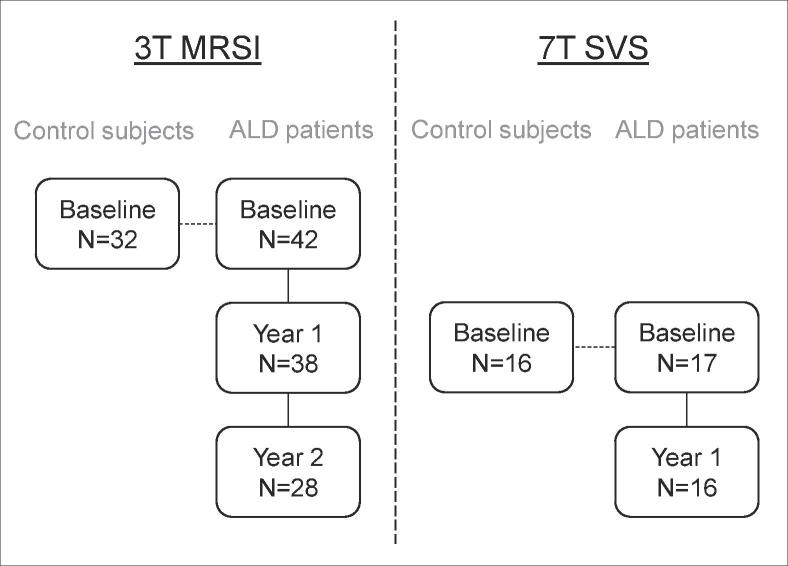
Study overview. An overview showing the timeline of included MRS scans per protocol: on the left 3T magnetic resonance spectroscopic imaging; on the right 7T single voxel spectroscopy, which was added to the imaging protocol one year later.

All 3T MRI examinations were performed on the same day as the clinical examinations. 7T imaging was added to the imaging protocol at a later stage, therefore only two instead of three 7T MRIs were available per patient. 7T imaging was performed on a separate day, as close to the second and third 3T MRI as possible. (Fig. 1)

#### 2.3.1 3T protocol

**Fig. 2:**
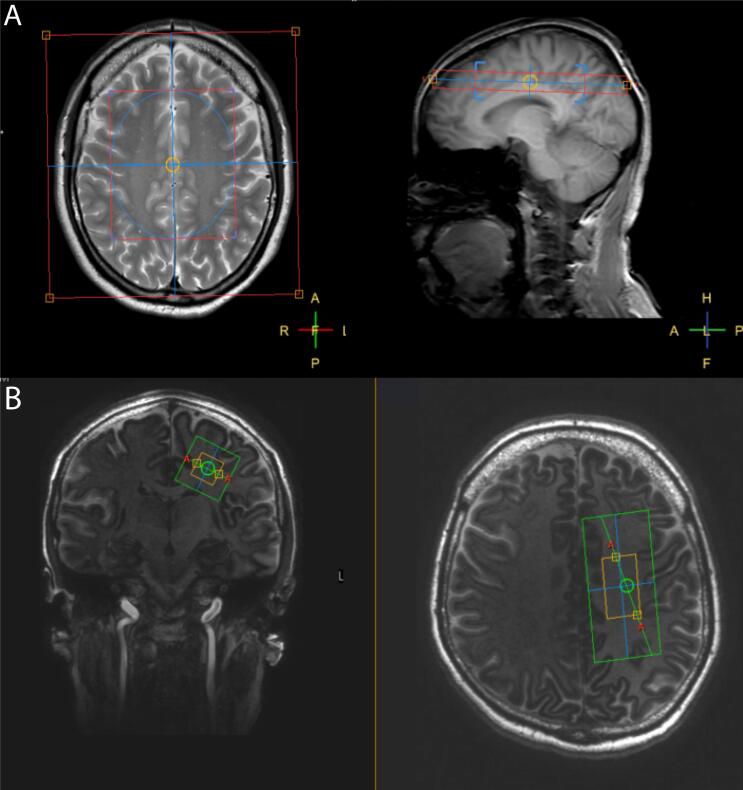
Slice and voxel placement. (A) 3T axial T2-weighted image of a healthy control subject showing placement of the MRSI slab. The VOI (small red rectangle, 80(RL) × 90(AP) mm) was centered one slice above the cerebrospinal fluid, onto the corpus callosum. Blue ellipsoid indicates the inner border of the rest slabs. (B) 7T MP2RAGE raw magnitude image of a healthy control subject showing placement of the single voxel (in yellow, 30(AP) × 15(RL) × 15(FH) mm) in the left and right sided semioval center. MRSI, magnetic resonance spectroscopic imaging; VOI, volume of interest; MP2RAGE, magnetization-prepared 2 rapid acquisition gradient echo; AP, anterior-posterior; RL, right-left; FH, feet-head. (For interpretation of the references to colour in this figure legend, the reader is referred to the web version of this article.)

Conventional MR imaging and MRSI were performed on a 3T MR scanner (Philips Ingenia; Philips Medical Systems, Best, Netherlands) using a 32-channel head coil. Three-dimensional T1-weighted fast field echo images (repetition time/echo time (TR/TE) = 7.6/3.5 ms; voxel size = 1.1x1.1x1.1 mm; field of view = 256x256x170 mm; number of slices = 155) and three-dimensional fluid attenuated inversion recovery (FLAIR) images were acquired (TR/inversion time (TI)/TE) = 4800/1650/356 ms; voxel size = 1.1x1.1x1.1 mm; field of view = 250x250x180 mm; number of slices = 160). MRSI was obtained with point resolved spectroscopy (PRESS) localization on a single axial slab (TR/TE 3000/35 ms; field of view 160 × 160 mm; volume of interest (VOI) 80x90 mm; 16x16 phase-encodings; BW = 1000; 1024 datapoints; NSA = 1; SENSE factor 2(AP)/2(RL), voxel size 10x10x15 mm) centered onto the corpus callosum*.* Outer volume suppression was performed using 10 circular rest slabs. (Fig. 2A) First and second order shimming was performed using FASTMAP. (Gruetter, 1993) Total acquisition time was 04:57 min.

#### 2.3.2 7T protocol

Conventional MR imaging and SVS were performed on a 7T MRI scanner (Philips Achieva; Philips Medical Systems, Best, Netherlands) with a volume transmit coil and a 32-channel receive coil (Nova Medical, Burlington, MA, USA). Magnetization-Prepared 2 Rapid Acquisition Gradient Echo (MP2RAGE) images were acquired for planning (TR/TE = 6.2/2.3 ms; voxel size = 0.64x0.64x0.64 mm; field of view = 220 × 220 × 164 mm; number of slices = 256). SVS was obtained using a semi-LASER sequence with FOCI pulses (Arteaga de Castro et al., 2013) (VOI size = 30(AP) × 15(RL) × 15(FH) mm; TR/TE = 5000/36 ms; BW = 4000; 2048 data points; NSA = 32; VAPOR water suppression (NSA = 2)). One VOI was planned in the left and one VOI in the right semioval center. (Fig. 2B) First and second order shimming was performed using FASTMAP. (Gruetter, 1993) Acquisition time was 02:50 min per voxel.

### 2.4 MRI data processing

#### 2.4.1 3T MRSI

**Fig. 3:**
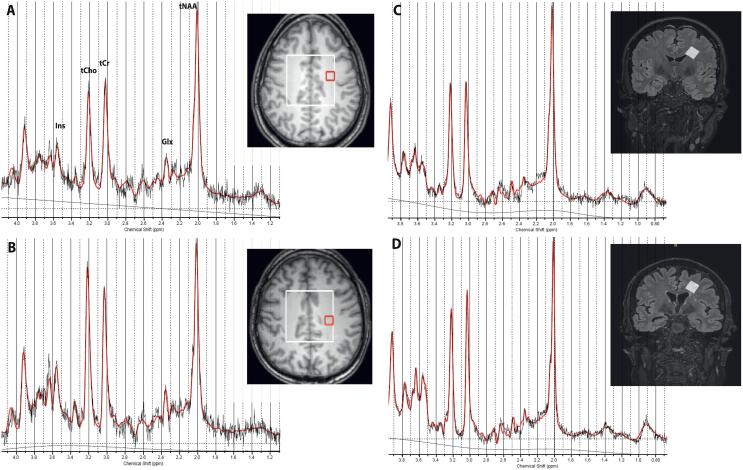
Quantification of spectra with LCModel. Left panel: the T1-weighted image was registered to the 3T MRSI slab; the white grid represents rows 6–11 and columns 6–11 that were considered for analysis. In red is the voxel from which the spectrum is displayed, (A) for a healthy control (FWHM 0.038 ppm, SNR 15) and (B) for a patient with moderate disease severity (FWHM 0.038 ppm, SNR 14). Right panel: the FLAIR image was registered to the 7T single voxel (in white). The spectrum is displayed, (C) for a healthy control (FWHM 0.039 ppm, SNR 24) and (D) for a patient with moderate disease severity (FWHM 0.033 ppm, SNR 25). MRSI, magnetic resonance spectroscopic imaging; FWHM, full width half maximum; SNR, signal-to-noise ratio. (For interpretation of the references to colour in this figure legend, the reader is referred to the web version of this article.)

Patients with typical cerebral ALD lesions were excluded, therefore lesion volume on FLAIR images was negligible and lesion segmentation and quantification was not performed. T1-weighted images were segmented into grey matter (GM), white matter (WM) and cerebrospinal fluid (CSF) partial volume estimates using SPM 12. The MRSI slab was registered to the T1 scan and the partial volume estimates using in-house Matlab scripts. Metabolites within the VOI were fitted between 1 and 4.2 ppm using a standard LCModel basis set for PRESS (TE = 35 ms) (Provencher, 1993) including simulated macromolecular contributions, with tCr as a reference metabolite (Fig. 3). Voxels were only included if they met the following spectral quality criteria: full width half maximum (FWHM) < 0.08 ppm, signal-to-noise ratio (SNR) > 8, Cramèr-Rao lower bounds (CRLB) < 20% for the sum of Glu and Gln (Glx). For the included voxels, for each of the estimated metabolites (tNAA, tCho, Ins and Glx), a non-linear least squares fit (Curve Fitting Toolbox, Matlab) was performed to estimate the mean metabolite ratios in GM and WM. In this analysis, metabolite concentrations in CSF were assumed negligible. FWHM and SNR values were extracted from LCModel.

#### 2.4.2 7T SVS

Metabolites were fitted between 0.5 and 4.0 ppm using LCModel with a simulated basis set (based on (Govindaraju et al., 2000) for sLASER (TE = 34 ms) with a measured macromolecular baseline (Fig. 3). Spectra were included for analysis if they met the following spectral quality criteria: FWHM < 0.08 ppm, SNR > 7 and CRLB (Glu) < 15%. The same metabolite concentration ratios were calculated as with 3T MRSI (tNAA, tCho, Ins and Glx) with tCr as a reference metabolite. Lac concentrations were too low to measure reliably and therefore excluded.

### 2.5 Statistical analysis

Data were summarized as means with standard deviations (SDs) or medians with interquartile ranges (IQR), depending on the distribution of the data. Normality of the data was assessed by visual inspection and Shapiro-Wilk test for normality.

Baseline differences in metabolite ratios between patients and controls were assessed using Student’s *t* test (normally distributed data). Effect sizes were described by measuring Cohen’s *d.* For 3T MRSI we also compared metabolite ratios between three groups: controls, asymptomatic patients and symptomatic patients, using one-way ANOVA with post-hoc analysis according to Tukey. Pearson’s *R* correlation coefficient (normally distributed data) or Spearman’s Rho (non-normally distributed data) was used to calculate the correlation between metabolite ratios and clinical measures. To correct for the influence of age on metabolite ratios, we used multiple linear regression analysis.

For 3T MRSI longitudinal analysis (three time-points) we used longitudinal mixed model analysis to assess differences in metabolite ratios over time and Kruskall Wallis test to assess differences in clinical outcomes between baseline and 2-year follow-up. For 7T SVS longitudinal analysis (two time-points) we used paired *t*-test to assess differences in clinical parameters and metabolite ratios over time. Differences between metabolite ratios acquired with 3T and 7T were assessed using Bland-Altman method comparison.

For all statistical tests a significance level of α = 0.05 (2-sided) was chosen. IBM SPSS Statistics Version 25 was used for statistical analysis.

## 3 Results

### 3.1 3T MRSI

Clinical characteristics of patients are described in Table 1. In summary, scores on clinical outcome measures indicated moderate disease severity. Thirty-one patients (70%) were scored as symptomatic. The mean age of patients (44.3 ± 16.2) and controls (42.4 ± 17.3) was comparable (p = 0.67).

**Table 1:** Baseline characteristics.

Clinical characteristics 3T MRSI	Control (n = 32)	Patient (n = 44)	Asymptomatic (n = 13)	Symptomatic (n = 31)
Age, yr	42.4 ± 17.3	44.3 ± 16.2	30.1 ± 13.0	50.2 ± 13.5
EDSS	–	3.5 (1.5–5.5)	1.0 (0.1–1.9)	4.5 (3.3–5.8)
SSPROM	–	86.5 (75.3–97.8)	100 (99–100)	79 (71.5–86.5)
Timed Up-and-Go (s)	–	5.3 (4.1–10.2)	3.7 (3.3–4.2)	8.7 (5.1–10.6)
Vibration score foot	–	2.8 (0.0–5.7)	7.3 (7.0–7.6)	1.8 (1.1–2.4)
Symptomatic myelopathy (%)	–	31 (70%)	–	–


Clinical characteristics 7T SVS	Control (n = 17)	Patient (n = 18)		
Age, yr	45.7 ± 16.4	46.5 ± 15.1		
EDSS	–	4.3 (2.9–5.8)		
SSPROM	–	85 ± 10.7		
Timed Up-and-Go (s)	–	7.8 ± 3.9		
Vibration score foot	–	1.1 (0.0–3.8)		
Symptomatic myelopathy (%)	–	14 (78%)		

Values are displayed as mean ± SD for normally distributed data and median (interquartile range) for non-normally distributed data.

EDSS, Expanded Disability Status Scale; SSPROM, Severity Scoring system for Progressive Myelopathy.

Baseline MRI, including MRSI was available for 44 adult patients. Two full MRSI datasets were excluded due to poor quality. The control group consisted of 32 participants, resulting in 32 MRSI datasets, of which none were excluded. Across all included voxels, median FWHM was 0.046 ppm and median SNR was 12.

#### 3.1.1 Between-group differences and clinical correlations

**Fig. 4:**
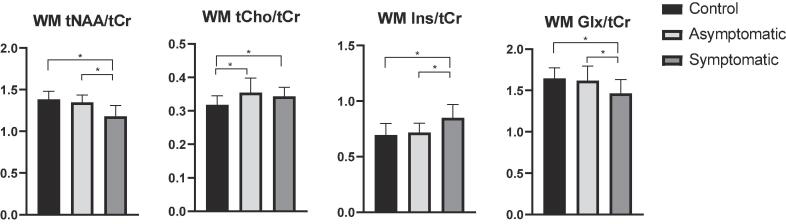
White matter metabolite concentration ratios per group. Bars show WM metabolite ratios measured with 3T MRSI for controls, asymptomatic and symptomatic patients. Differences between groups were analyzed using one-way ANOVA with Tukey’s post-hoc analysis. Stars indicate significant differences between groups. WM, white matter; tNAA, N-acetylaspartate; tCho, choline containing compounds; Ins, inositol; Glx, glutamate and glutamine combined.

All baseline metabolite ratios measured in the brain WM differed significantly between patients and controls (Table 2). Patients had lower tNAA/tCr and Glx/tCr and higher tCho/tCr and Ins/tCr compared to controls. In the GM, only tNAA/tCr and Glx/tCr differed significantly between groups, and were lower in patients compared to controls. When stratifying patients based on disease severity (asymptomatic versus symptomatic patients), we found that differences in WM metabolite ratios were almost completely explained by the symptomatic subgroup; except for tCho, for which significant differences were found between controls and both patient groups, but not between asymptomatic and symptomatic patients (Fig. 4).

**Table 2:** Baseline metabolite ratio’s.

Baseline metabolite ratios 3T MRSI	Control (n = 32)	Patient (n = 42)	Mean difference (95% CI)	p-value	Effect size (D)
WM tNAA/tCr	1.38 ± 0.10	1.23 ± 0.14	−0.15 (−0.21 to −0.09)	<0.001	1.23
WM tCho/tCr	0.32 ± 0.03	0.35 ± 0.03	0.03 (0.01 to 0.04)	<0.001	1.00
WM Ins/tCr	0.70 ± 0.10	0.81 ± 0.12	0.11 (0.06 to 0.17)	<0.001	0.99
WM Glx/tCr	1.65 ± 0.13	1.51 ± 0.18	−0.13 (−0.21 to −0.06)	0.001	0.89
GM tNAA/tCr	1.01 ± 0.09	0.96 ± 0.10	−0.05 (−0.10 to 0.01)	0.029	0.53
GM tCho/tCr	0.22 ± 0.02	0.21 ± 0.02	−0.01 (−0.02 to 0.00)	0.191	0.50
GM Ins/tCr	0.60 ± 0.08	0.57 ± 0.11	−0.03 (−0.08 to 0.01)	0.150	0.31
GM Glx/tCr	2.19 ± 0.21	2.03 ± 0.21	−0.16 (−0.27 to −0.04)	0.009	0.76


Baseline metabolite ratios 7T SVS	Control (n = 16)	Patient (n = 18)	Mean difference (95% CI)	p-value	Effect size (D)
WM tNAA/tCr	1.75 ± 0.16	1.51 ± 0.21	−0.25 (−0.37 to −0.12)	<0.001	1.29
WM tCho/tCr	0.31 ± 0.03	0.34 ± 0.04	0.03 (0.01 to 0.06)	0.006	0.85
WM Ins/tCr	0.78 ± 0.10	0.99 ± 0.17	0.22 (0.11 to 0.32)	<0.001	1.51
WM Glx/tCr	1.00 ± 0.13	0.87 ± 0.12	− 0.13 (−0.22 to −0.05)	0.004	1.03

Values are displayed as mean ± SDs.

Differences between groups are analyzed with Student's *t*-test.

WM, white matter; GM, grey matter; tNAA, N-acetylaspartate; tCho, choline containing compounds; Ins, inositol; Glx, glutamate + glutamine; tCr, creatine.

**Fig. 5:**
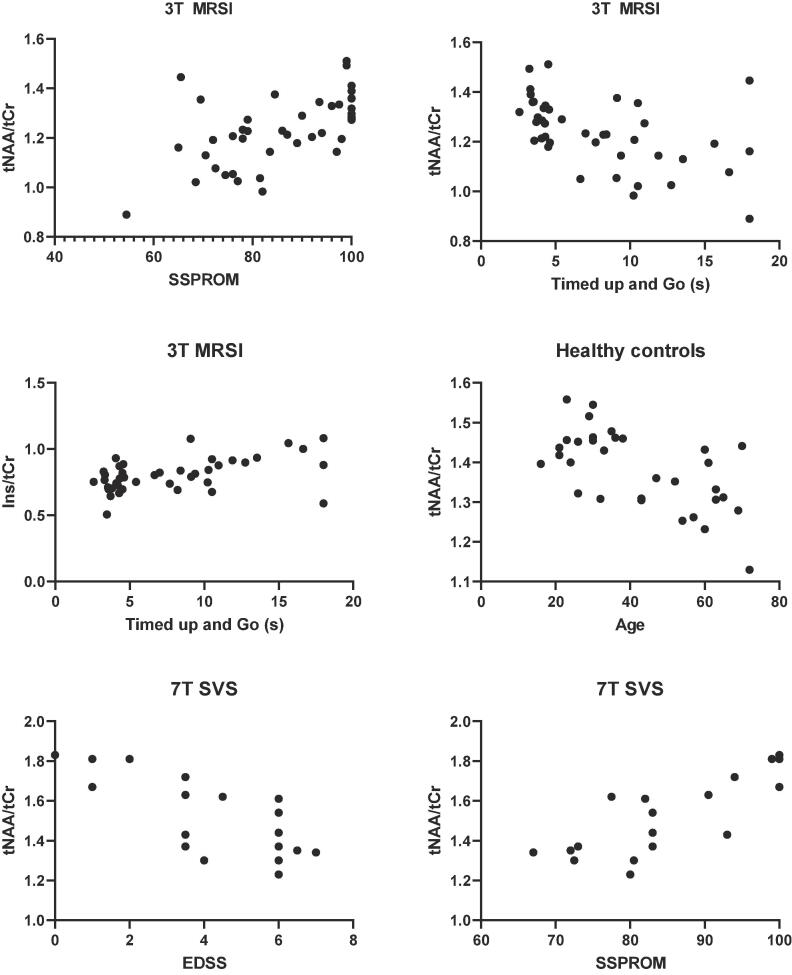
Associations between clinical parameters and metabolite concentration ratios. Scatterplots show associations between clinical outcome measures and 3T MRSI WM metabolite ratios, for (A) SSPROM and tNAA/tCr: r = 0.58p=<0.001; (B) TUG and tNAA/tCr: r = -0.64p=<0.001; (C) TUG and Ins/tCr: r = 0.54p = 0.001 and associations between clinical outcome measures and 7T SVS WM metabolite ratios for (E) EDSS and tNAA/tCr: r = -0.747p=<0.001; (F) SSPROM and tNAA/tCr: r = 0.771p=<0.001. (D) association between age and WM tNAA/tCr in healthy controls: r = -0.62p=<0.001. MRSI, magnetic resonance spectroscopic imaging; WM, white matter; SSPROM, severity scoring system for progressive myelopathy; EDSS, expanded disability status scale; TUG, timed up and go; tNAA, N-acetylaspartate; tCr, creatine; Ins, inositol; SVS, single voxel spectroscopy.

All clinical measures showed significant correlations with metabolite ratios (Fig. 5). Age was a significant predictor for all metabolite ratios. After correction for age, we found a significant predictive value for SSPROM (B = 0.31, p = 0.021) and TUG (B = −0.32, p = 0.03) on WM tNAA/tCr and for TUG on WM Ins/tCr (B = 0.31, p = 0.03).

#### 3.1.2 Disease progression

Follow-up scans were available for 38 (year 1) and 28 (year 2) patients. The median time between scans was 11 months from baseline to year 1 and from year 1 to year 2. During the entire follow-up only one patient converted from asymptomatic to symptomatic status. All clinical outcome measures, except TUG, could detect disease progression over the 2-year follow-up period. None of the metabolite ratios changed significantly over time. (Supplementary Table 1)

### 3.2 7T SVS

Clinical characteristics were similar to the 3T MRSI group (Table 1). The mean age of patients (46.5 ± 15.1) and controls (45.7 ± 16.4) was comparable (p = 0.88).

A total of 18 patients underwent 7T imaging twice, resulting in 72 acquired spectra of which 65 were included. Seven spectra were excluded due to poor quality, amongst which three right sided and four left sided spectra. In the control group a total 17 subjects underwent 7T imaging once, resulting in 34 acquired spectra. Three spectra were excluded due to poor quality, of which two spectra belonged to the same subject. For all spectra combined, median FWHM was 0.046 ppm and median SNR was 21. The tissue fraction (GM/WM/CSF) in healthy controls and patients was comparable (0.08/0.91/0.01 versus 0.04/0.96/0.00).

#### 3.2.1 Between-group differences and clinical correlations

All measured baseline metabolite ratios differed significantly between patients and controls. Patients had lower tNAA/tCr and Glx/tCr and higher tCho/tCr and Ins/tCr (Table 2). Due to the small sample size, we were not able to stratify patients into groups based on their symptomatic status.

Significant correlations between metabolite ratios and clinical measures were found between tNAA/tCr and EDSS, SSPROM and vibration score foot and between Ins/tCr and EDSS, SSPROM and vibration score foot (Fig. 5). There were no correlations between tCho/tCr or Glx/tCr and clinical outcome measures. Age was a significant predictor for all metabolite ratios. After correction for age, we found a significant predictive value for SSPROM (B = 0.01, p = 0.006) and EDSS (B = -0.05, p = 0.026) on tNAA/tCr and for SSPROM on Ins/tCr (B = -0.01, p = 0.022).

#### 3.2.2 1-year follow-up

Follow-up scans were available for 16 patients. The median time between baseline and follow-up scan was 9 months (IQR 9–10). From the clinical outcome measures only SSPROM was able to detect disease progression after 1 year (mean change −5.2, *p =* 0.004). There was no difference in metabolite ratios between baseline and 1-year follow-up (p > 0.05). (Supplementary Table 2)

### 3.3 Comparisons between 3T and 7T imaging methods

When comparing metabolite ratios between 3T MRSI and 7T SVS we found a bias of −0.006 ± 0.02 (95% limits of agreement from −0.05 to 0.03) for the tCho/tCr levels. The bias for ratio of the difference versus the average was 0.98 ± 0.06 (95% limits of agreement from 0.86 to 1.10) (Supplementary Fig. 1). Similar results were found for the other metabolite ratios.

## 4 Discussion

In this prospective imaging study, we showed that brain metabolite levels of adult ALD patients without cerebral involvement, measured with 3T MRSI and 7T SVS, have the potential to be utilized as imaging biomarkers in ALD. Metabolite ratios differed between clinically distinct groups and correlate with severity of myelopathy. Although clinical measures demonstrated progression of myelopathy, brain metabolite ratios remained unchanged during the two-year follow-up period.

In adult ALD patients neurodegeneration occurs predominantly in the corticospinal tracts and dorsal columns of the spinal cord and this axonal degeneration is the pathological hallmark of the myelopathy in ALD (Powers et al., 2000). Recently, it has been shown that even in cerebral ALD extensive axonal degeneration occurs primarily, followed by a loss of myelin (Bergner et al., 2021). tNAA and Glu both are markers for axonal damage, and reduced tNAA levels are found in almost all neurodegenerative processes. In the present study, symptomatic patients had significantly lower WM tNAA/tCr and Glx/tCr, compared to controls, corresponding to previous MRS studies in ALD (Pouwels et al., 1998, Dubey et al., 2005, Eichler et al., 2002, Ratai et al., 2008). With the 7T data it was possible to separate Glu and Gln and we observed that the lower Glx/tCr in patients was driven by a decrease in Glu, as Gln was not different between patients and controls (data not shown). Moreover, GM tNAA/tCr and Glx/tCr were also lower compared to controls. Decreased tNAA in the cortex of ALD patients has been described before (Ratai et al., 2008); however, a decrease in cortical Glx/tCr is a novel finding. Our results show that neuronal loss is present throughout the brain – including the GM – of ALD patients and is not confined to the spinal cord and peripheral nerves. Another novel finding is the elevation of Ins/tCr in patients, even in the absence of active inflammation or demyelination. Elevated Ins is generally considered a marker for gliosis (Pouwels et al., 1998, Öz et al., 2014). In the two previous ALD studies that assessed Ins, significantly increased Ins levels were found only in pediatric and adult patients with active cerebral ALD (Pouwels et al., 1998, Ratai et al., 2008), but not in asymptomatic patients or males and females with myelopathy. Our findings suggest that a mild elevation of Ins, as found in this study, is already present in patients without cerebral ALD. In addition to Ins, higher tCho levels are associated with gliosis and indicate processes of active demyelination, inflammation or cell proliferation (Mader et al., 2008). In ALD, increased tCho levels have been described in adult and pediatric patients with cerebral ALD (Pouwels et al., 1998, Ratai et al., 2008). In males and females with myelopathy reports are ambiguous, as one study showed no significant elevation of tCho/tCr and another study showed significant elevation only in the frontal white matter (Fatemi et al., 2003, Dubey et al., 2005). In this study, we excluded patients with cerebral ALD and nevertheless found a significant elevation in tCho/tCr in patients. This elevation is probably not a result of demyelination or inflammation as these pathological processes are not ongoing in ALD patients with myelopathy (Schaumburg et al., 1977, Engelen et al., 2014). Furthermore, the tCho/tCr ratio was the only metabolite ratio that differed between asymptomatic patients and controls but not between asymptomatic and symptomatic patients. This suggests that tCho levels are increased in all types of ALD patients, even before symptoms of a myelopathy are present, and may be an early marker for neurodegeneration.

We found the strongest correlations, after adjustment for age, between measures of severity of myelopathy and tNAA/tCr ratios. This confirms that tNAA levels are indeed specific for neuroaxonal damage and are associated with clinical disability. tNAA/tCr ratios may therefore be of potential value as biomarker for severity of myelopathy in ALD. We also found correlations between Ins/tCr and TUG, which has been described before (Ratai et al., 2008). However, these correlations are measured indirectly, and biomarkers measured directly in the spinal cord – for example magnetization transfer imaging or diffusion tensor imaging (Huffnagel et al., 2019, Fatemi et al., 2005) – may be even more valuable.

Longitudinally, there were no significant changes in metabolite levels. This is probably due to the slow progression of disease in ALD, in which symptoms progress over years to decades. (Huffnagel et al., 2019) Our findings show that neither 3T MRSI nor 7T SVS was sensitive enough to measure disease progression in ALD in a relatively short follow-up, whereas clinical measures were.

When comparing the two MRS methods, we found that both imaging methods produce similar results (Supplementary Fig. 1**)**. In the 7T SVS group we found larger effect sizes for differences between patients and controls for almost all of the metabolite ratios. Additionally, SNR in the 7T SVS group was higher. However, it should be noted that direct comparisons between field strengths are difficult, because of differences in localization methods, timing parameters, field inhomogeneities and relaxation times, and this was not the primary aim of this study.

A limitation of this study is, first, that the follow-up period was relatively short, making it difficult to conclude on the predictive value of MRS on disease progression. Second, the number of available 7T scans was limited as many patients were not eligible or not motivated to participate and we had to exclude a substantial number of spectra in this 7T subgroup, due to poor quality. The quality of the 7T scans may have been compromised due to an accidentally large shim box in some of the scans. Although this may have affected the linewidth of our spectra, the quality of the data was sufficient for metabolite quantification.

We can conclude that, in addition to providing pathophysiological insights into the disease, MRS – and more specifically the tNAA/tCr ratio – is of potential value as imaging biomarker for neurodegeneration in ALD. It should however be noted that the added value to the currently available clinical outcome measures for monitoring disease progression has not been shown. In this homogenous group of ALD males without cerebral involvement we could not draw conclusions on the value of MRS in predicting disease course. However, MRS may play a promising role in cohorts in which disease course changes, for example in predicting the onset of cerebral ALD or in providing insight into the pathophysiological changes after hematopoietic stem cell transplant. These hypotheses will be evaluated in future studies of our ALD cohort.

## 5 Declarations

### 5.1 Ethics approval

The study protocol was approved by the local Institutional Review Board (METC 2014_347). All participants (and/or their legal guardians) gave written informed consent prior to participation.

### 5.2 Consent for publication

Not applicable.

### 5.3 Availability of data and materials

The datasets used an/or analyzed during the current study are available from the corresponding author on reasonable request.

### CRediT authorship contribution statement

**Stephanie I.W. van de Stadt:** Conceptualization; Formal analysis; Validation; Writing – original draft. **Anouk Schrantee:** Formal analysis; Software; Validation; Writing – review & editing. **Irene C. Huffnagel:** Conceptualization; Investigation; Writing – review & editing. **Wouter J.C. van Ballegoij:** Conceptualization; Investigation; Writing – review & editing. **Matthan W.A. Caan:** Resources; Software; Writing – review & editing. **Petra J.W. Pouwels:** Software; Supervision; Validation; Writing – review & editing. **Marc Engelen:** Conceptualization; Funding acquisition; Supervision; Writing – review & editing.

## Funding

This work was supported by a grant from the Netherlands Organization for Scientific Research (VENI grant: 016.156.033 to M.E. and VIDI grant: 016.196.310 to M.E.).

## Declaration of Competing Interest

The authors declare that they have no known competing financial interests or personal relationships that could have appeared to influence the work reported in this paper.
