# Supplementary figures and images for: Magnetic resonance spectroscopy as marker for neurodegeneration in X-linked adrenoleukodystrophy
Source: Neuroimage Clin. 2021 Aug 24;32:102793. doi: 10.1016/j.nicl.2021.102793 (PMC8405970; doi:10.1016/j.nicl.2021.102793)

# Bland-Altman of 3T MRSI and 7T SVS

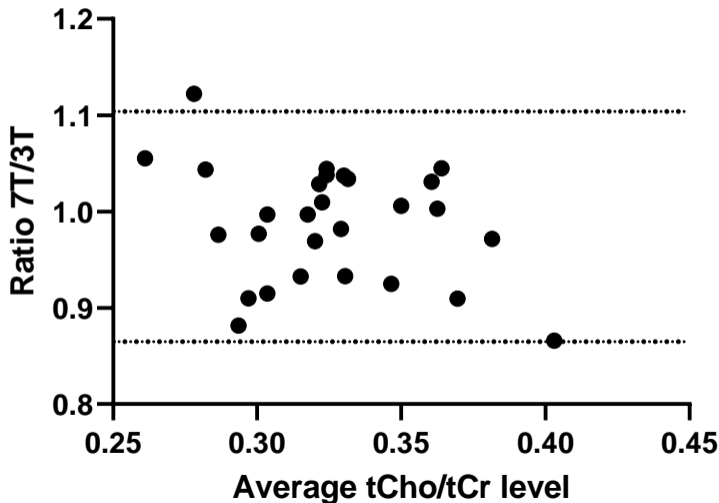

Supplement: Supplementary data 1 [file mmc1.pdf]
