# Supplementary material for: Magnetic resonance spectroscopy as marker for neurodegeneration in X-linked adrenoleukodystrophy
Source: Neuroimage Clin. 2021 Aug 24;32:102793. doi: 10.1016/j.nicl.2021.102793 (PMC8405970; doi:10.1016/j.nicl.2021.102793)
Supplement: Supplementary data 2 [file mmc2.docx]

**Supplementary Figure 1.**

Bland-Altman plot of the 3T MRSI method versus the 7T SVS method showing the average tCho/tCr concentration on the x-axis versus the ratio of 7T/3T acquired tCho/tCr metabolite levels on the y-axis. 95% limits of agreement are shown with dotted lines (0.86 to 1.10).

MRSI, magnetic resonance spectroscopic imaging; SVS, single voxel spectroscopy; tCho, choline; tCr, creatine.

**Supplementary Table 1. Longitudinal 3T MRSI analysis**

| **Metabolite ratios** | **Baseline (n=42)** | **Year 1 (n=38)** | **Year 2 (n=28)** | **Mean difference (95% CI)  Baseline – Year 2** | **p-value** |
| --- | --- | --- | --- | --- | --- |
| WM tNAA/tCr | 1.23 ± 0.14 | 1.21 ± 0.25 | 1.22 ± 0.15 | -0.01 (-0.03 to 0.02) | 0.823 |
| WM tCho/tCr | 0.35 ± 0.03 | 0.33 ± 0.06 | 0.33 ± 0.03 | -0.01 (-0.02 to -0.01) | 0.140 |
| WM Ins/tCr | 0.81 ± 0.12 | 0.78 ± 0.18 | 0.81 ± 0.13 | 0.00 (-0.03 to 0.03) | 0.625 |
| WM Glx/tCr | 1.51 ± 0.18 | 1.45 ± 0.28 | 1.54 ± 0.19 | 0.02 (-0.09 to 0.05) | 0.236 |
|  |  |  |  |  |  |
|  |  |  |  |  |  |
| **Disease progression** | **Baseline  (n=32)** | **Year 2  (n=32)** | **Mean difference (95% CI)** | **p-value** |  |
| EDSS | 3.5 (1.3-5.7) | 4.0 (2.1-6.0) | 0.33 (0.08 to 0.58) | 0.01 |  |
| SSPROM | 85.3 (73.4-97.3) | 83.0 (69.1-97.0) | -2.1 (-3.8 to -0.3) | 0.024 |  |
| Timed Up-and-Go (s) | 4.5 (1.8-7.2) | 5.4 (2.6-8.2) | 0.32 (-0.1 to 0.75) | 0.182 |  |
| Vibration score foot | 3.8 (0.9-6.6) | 3.4 (0.3-6.5) | -0.47 (-0.81 to -0.13) | 0.008 |  |
|  |  |  |  |  |  |
| Values are displayed as mean ± SD or median (interquartile ranges) | | |  |  |  |
| Differences between groups are analyzed with Kruskal Wallis test or longitudinal mixed models | | | |  |  |

**Supplementary Table 2. Longitudinal 7T SVS analysis**

| **Metabolite ratios follow-up** | **Baseline (n=16)** | **Year 1 (n=16)** | **Difference (95% CI)** | **p-value** |
| --- | --- | --- | --- | --- |
| WM tNAA/tCr | 1.49 ± 0.20 | 1.46 ± 0.19 | -0.03 (-0.07 to 0.01) | 0.109 |
| WM Ins/tCr | 0.34 ± 0.04 | 0.34 ± 0.03 | 0.00 (-0.01 to 0.01) | 0.688 |
| WM tCho/tCr | 0.98 ± 0.17 | 1.02 ± 0.19 | 0.04 (-0.02 to 0.10) | 0.161 |
| WM Glx/tCr | 0.87 ± 0.12 | 0.86 ± 0.12 | -0.01 (-0.07 to 0.05) | 0.740 |
|  |  |  |  |  |
| **Disease progression** | **Baseline (n=18)** | **Year 1 (n=18)** | **Difference (95% CI)** | **p-value** |
| EDSS | 4.2 ± 2.1 | 4.4 ± 2.1 | 0.19 (-0.1 to 0.5) | 0.109 |
| SSPROM | 85.0 ± 10.7 | 79.8 ± 14.7 | -5.2 (-8.5 to -1.9) | 0.004 |
| Timed Up-and-Go (s) | 7.8 ± 3.9 | 8.0 ± 3.8 | 0.25 (-0.5 to 1.0) | 0.148 |
| Vibration score foot | 1.1 (0.0-3.8) | 0.9 (0.0-3.4) | -0.12 (-0.47 to 0.22) | 0.463 |
|  |  |  |  |  |
| Values are displayed as mean ± SD or median (interquartile ranges) |  |  |  |  |
| Differences between groups are analyzed with Paired t-test or Wilcoxon signed rank test | |  |  |  |
|  | | | |  |
